# Tongue Abnormalities Are Associated to a Maternal Folic Acid Deficient Diet in Mice

**DOI:** 10.3390/nu10010026

**Published:** 2017-12-28

**Authors:** Estela Maldonado, Yamila López-Gordillo, Teresa Partearroyo, Gregorio Varela-Moreiras, Concepción Martínez-Álvarez, Juliana Pérez-Miguelsanz

**Affiliations:** 1 Laboratorio de Desarrollo y Crecimiento Craneofacial, Facultad de Odontología, Universidad Complutense de Madrid, 28040 Madrid, Spain; estemalba@yahoo.es (E.M.); yamila.bio165@gmail.com (Y.L.-G.); cmartinz@ucm.es (C.M.-Á.); 2 Departamento de Ciencias Farmacéuticas y de la Salud, Facultad de Farmacia, Universidad CEU San Pablo, Boadilla del Monte, 28003 Madrid, Spain; t.partearroyo@ceu.es (T.P.); gvarela@ceu.es (G.V.-M.); 3 Departamento de Anatomía y Embriología Humana, Facultad de Medicina, Universidad Complutense de Madrid, 28040 Madrid, Spain

**Keywords:** tongue development, head development, congenital abnormalities, aglossia, microglossia, maternal folic acid-deficient diet, C57 mouse

## Abstract

It is widely accepted that maternal folic acid (FA) deficiency during pregnancy is a risk factor for abnormal development. The tongue, with multiple genes working together in a coordinated cascade in time and place, has emerged as a target organ for testing the effect of FA during development. A FA-deficient (FAD) diet was administered to eight-week-old C57/BL/6J mouse females for 2–16 weeks. Pregnant dams were sacrificed at gestational day 17 (E17). The tongues and heads of 15 control and 210 experimental fetuses were studied. In the tongues, the maximum width, base width, height and area were compared with width, height and area of the head. All measurements decreased from 10% to 38% with increasing number of weeks on maternal FAD diet. Decreased head and tongue areas showed a harmonic reduction (Spearman nonparametric correlation, Rho = 0.802) with respect to weeks on a maternal FAD diet. Tongue congenital abnormalities showed a 10.9% prevalence, divided in aglossia (3.3%) and microglossia (7.6%), always accompanied by agnathia (5.6%) or micrognathia (5.2%). This is the first time that tongue alterations have been related experimentally to maternal FAD diet in mice. We propose that the tongue should be included in the list of FA-sensitive birth defect organs due to its relevance in several key food and nutrition processes.

## 1. Introduction

Craniofacial development requires extremely fine-tuned developmental coordination of multiple specialized tissues, such as the surface ectoderm, neural crest, mesoderm, and the pharyngeal endoderm, which provides protection and functional integration [1,2].

The tongue is a complex, partially mobile organ which comprises eight bilateral muscles incompletely separated by a median septum. It is covered by a mucosa, which carries taste buds and is innervated by five cranial nerves [1,2]. It is involved in several important physiological tasks, such as mastication, tasting, swallowing and human speech [1,2].

The tongue is derived from swellings on the floor of the all branchial arches, beginning its development at the end of the fourth week in humans and at embryonic day (E) 10.5 in mice. The cell origin of the tongue is hybrid. Both the connective tissue and vasculature derive from the cranial neural crest (NC) cells from the embryonic midbrain and the rostral first and second rhombomers of the hindbrain [2,3,4,5,6]. Most of the tongue muscles originate from myogenic progenitors from occipital somites 2 to 5. Cranial paraxial mesoderm contributes to the formation of the exterior tongue muscles [2,3,4,5,6].

In mouse embryos, the cranial NC cells populate the tongue primordium before the invasion of myogenic progenitors. Cranial NC cells are not required for myogenic progenitor migration, however, when they first enter the craniofacial region, an immediate intimate contact between the two cell types is established, which continues throughout the entire course of tongue morphogenesis. The intimate relationship between these cell lineages suggests that reciprocal interactions between cranial NC cells and myogenic cells may occur during tongue development [2,5,6].

More than 80% of congenital malformations have a complex etiology, in which interactions between subtle structural genetic and environmental exposures such as periconceptional malnutrition and unhealthy lifestyles are implicated [7]. In the tongue, multiple factors such as the Wnt signaling pathway, transforming growth factor beta (TGF-β) and bone morphogenetic proteins (BMPs) regulate migration, pattering, proliferation, determination, differentiation and maturation either in different subpopulations of cranial NC cells or in migrating myogenic progenitors [3,5,6,7,8,9,10]. Environmental factors may alter this essential balance for normal development, i.e., excess retinoic acid is a known teratogen that causes malformations of the tongue and the palate in both human and rodents [11].

Folic acid (FA) is one of the clearest examples of the influence of the environment on development. Folate is vital for fetal development [12,13,14,15]. FA deficiency causes errors in DNA synthesis as well as hypomethylation and hyperhomocysteinemia, and inhibits cell growth and programmed cell death [16]. Folate deficiency has been directly related to neural tube defects (NTD), orofacial development, cardiovascular disease risk, cancer, and mental illness [10,16,17,18,19]. Interestingly, FA deficiency causes cleft palate in mice and humans, a congenital malformation affecting the first branchial arch, as previously shown [20,21]. Mechanisms whereby periconceptional folate influences normal development and disease are still poorly understood, and epigenetics may be involved in newborn humans [22,23,24].

The tongue, with multiple genes acting together in a coordinated cascade with respect to time and position, has emerged as a target organ for testing the effect that FA could exercise during development. Thus, for the first time we report the influence of maternal FAD diet on tongue development and propose that the tongue be added to the list of FA-sensitive birth defect organs.

## 2. Materials and Methods

### 2.1. Animals and Diets

A total of 57 eight-week-old C57/BL/6J female mice (Harlan Laboratories, Barcelona, Spain) were divided in two groups based on the experimental diet administered, of which only macro and microscopic analysis were performed 43 and 41, respectively. The diets were adjusted to mice requirements, and were based on a pure amino acid diet (Harlan Laboratories, Inc., Indianapolis, IN, USA), only modified in its FA content as follows: control diet group (2 mg FA/kg diet, SAFE-DIETS A04/03, Panlab) and maternal FA-deficient (FAD) diet group (0 mg FA/kg diet + 1% succinylsulfathiazole, Harlan Laboratories, TD02490). Animals were maintained in a 12:12 h dark/light cycle, under controlled temperature and humidity conditions at the animal care unit of the Facultad de Medicina of the Universidad Complutense (Madrid, Spain). Mice were fed their respective diets *ad libitum* for 2, 4, 6, 8, 10, 12, 14 or 16 weeks and were immediately euthanized. These diets have been already successfully used in previous studies by our research group [21,25]. Manipulation of the animals was performed following the European Union Normative (2003/65/CE). The experimental protocol used was reviewed and ethically approved by the Animal Experimentation Committee of the Universidad Complutense of Madrid.

For folate levels analysis, livers from pregnant females on the control (*n* = 9) or FAD diet for 2 (*n* = 8), 4 (*n* = 7), 6 (*n* = 4), 8 (*n* = 12), 10 (*n* = 10), 12 (*n* = 5), 14 (*n* = 2) and 16 (*n* = 4) weeks were collected and processed as previously reported [21,25].

### 2.2. Morphological Study

At gestation day 17, female mice were killed by cervical dislocation, and fetuses were removed by caesarean section, placed in cold phosphate-buffered saline and processed as previously described [16,18,23]. Briefly, fetuses were externally examined for the presence of macroscopic malformations using a Nikon C-DS microscope (Nikon Corp., Tokyo, Japan) and a Leica EC3 digital camera (Leica Geosystems AG, St. Gallen, Switzerland). Heads were separated from bodies to facilitate microtome cutting. Samples were fixed in 10% buffered formaldehyde solution, dehydrated, embedded in paraffin, and 7-μm serial coronal sections were obtained. Sections were stained with hematoxylin-eosin following standard procedures and photographed using Leica DMR microscope (Leica) and a Leica DFC 320 digital camera (Leica). Both the head and tongue were further studied.

### 2.3. Tongue and Head Measurements

Comparable coronal sections of heads from control and experimental fetuses were selected as described below. Measures were taken using the image processing program Image J (National Institutes of Health (NIH), Bethesda, MD, USA, Department of Health and Human Services, Washington, DC, USA).

**Figure 1:**
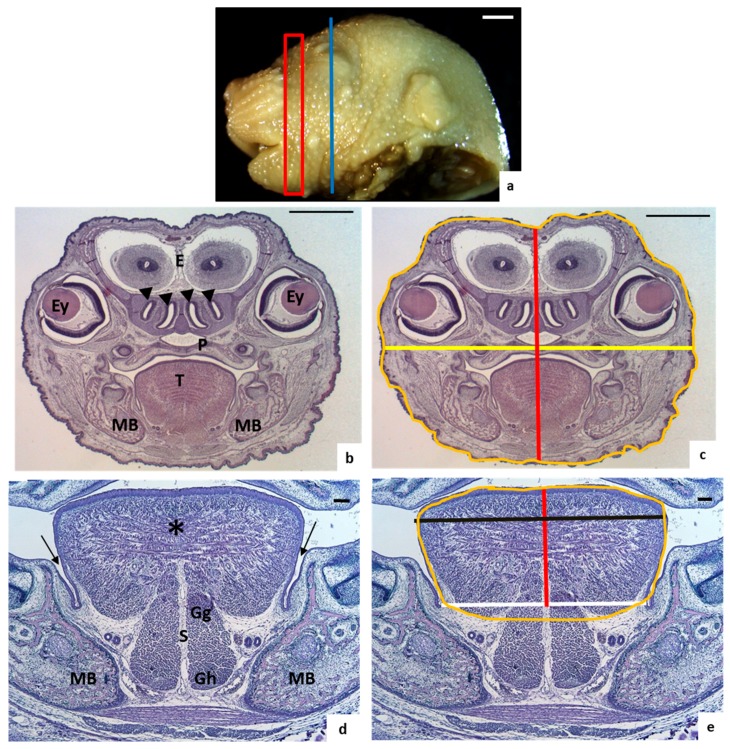
Tongue and head measurements. (**a**) Lateral view of the control fetus head. The red square marks the bloc of sections selected for tongue measurements. The blue line crossing the eye indicates the chosen region for head measurements. Scale bar: 1 mm; (**b**) Coronal section of a control head showing the references for head measurements: the nasal conchae (arrowheads) and the eyes (E: encephalon; Ey: eye; MB: mandibular bone; P: palate; T: tongue). Scale bar: 0.5 mm; (**c**) Description of head measures: yellow line for head width, red line for head height and orange line for head area; (**d**) Control section showing the tongue body with intrinsic muscles (asterisk), lingual septum (S), the lingual-mandibular sulcus (black arrows), mandibular bone (MB) and genioglossus (Gg) and geniohyoid (Gh) muscles. Scale bar: 100 µm; (**e**) Description of tongue measurements: black line for width of the widest zone of the tongue, red line for height, white line for width of the base of the tongue, and orange line for area. Scale bar: 100 µm.

For head measures, sections were chosen where two nasal conchae and the eyes were visible (Figure 1a–c). The height measurement of the head was obtained drawing a vertical line in the middle of the section, from the top to the bottom (Figure 1c), whilst the width measurement was taken drawing a horizontal line crossing the widest part of the palate (Figure 1c). These sections were posterior to the tongue sections in order to involve the important structures of the head such as the eyes and not only the nose of the animal.

For the measurement of the width of the base of the tongue, a line was drawn between the lingual-mandible grooves (Figure 1e). From this line to the top of the tongue, another line was drawn to measure the height of the tongue (Figure 1e). The widest part of the tongue was also measured (Figure 1e).

To measure the tongue and head areas, both structures were outlined (Figure 1c,e). Three continuous sections from each embryo were measured in all cases.

### 2.4. Statistical Analysis

Values are expressed as median (interquartile range) per group. Variables were tested for normality using a Shapiro–Wilk test. Statistical differences between cases and control groups were analysed by Kruskal–Wallis Test and the Dunn to adjust for multiple comparison and adjust the *p* value with Bonferroni correction, and Fisher’s exact test to find whether the proportions of mothers with tongue malformation fetuses are different from values of mothers without tongue malformation fetuses. Differences were considered significant at *p* < 0.05.

Spearman nonparametric correlation (Rho) was used for the total sample by variables. Correlation levels were classified according to the following categories: poor (Rho < 0.00), light (Rho = 0.00–0.20), fair (Rho = 0.21–0.40), moderate (Rho = 0.41–0.60), good (Rho = 0.61–0.80) and practically perfect (Rho = 0.81–1.00) [26]. All analysis were performed using the SPSS v.24.0 program (IBM Corp., Armonk, NY, USA).

## 3. Results

Maternal hepatic folate concentrations indicate the usefulness of our experimental model, as we have previously demonstrated [21,25]. The mean control folate value was 36.5 µg/g. After two and four weeks on the FAD diet, folate values were equal, and approximately half of the control (statistically significantly lower, *p* < 0.01). Folate values for the FAD diet groups over the period from six to 16 weeks were also statistically equal (5.2 µg/g on average) and significantly lower (*p* < 0.01) than for the 2–4 weeks FAD diet animals and the control group.

### 3.1. Morphological Analysis

A total of 235 fetal heads from control (*n* = 25) and experimental fetuses (*n* = 210) were externally analyzed for the presence of macroscopic malformations (Table 1 and Table 2, and Supplementary data). Control and 2–4-week maternal FAD fetuses did not show malformations in the tongue and/or mandible. However, 23 (9.8% of total) of the 6–16-week maternal FAD diet fetuses showed dysmorphologies in the tongue and mandible. In the 6–10-week maternal FAD diet fetuses, the number of individuals affected increased with the number of weeks under the maternal FAD diet.

**Figure 2:**
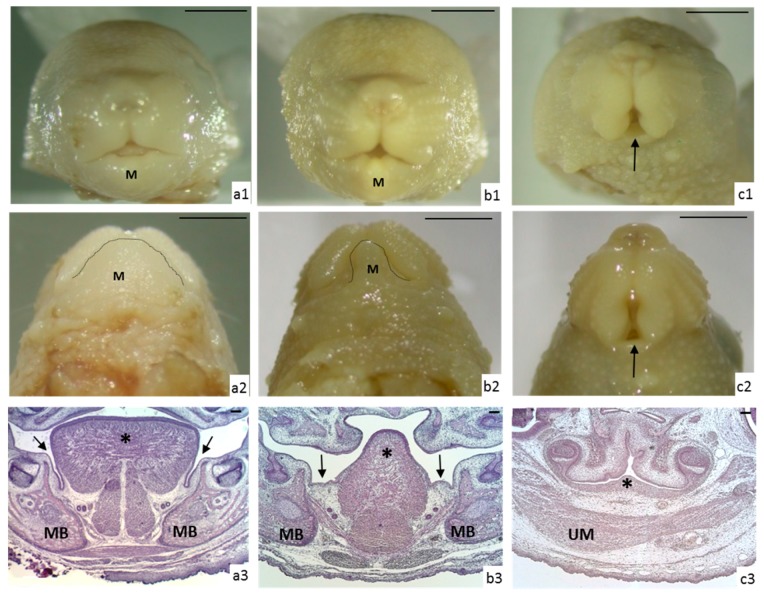
Morphological study of the tongue and head. (**a**) Control fetus: (a1) Frontal view of the head showing a triangular mouth (M: mandible); (a2) Inferior view showing the mandible (M) and a wide arcade (dotted line); (a3) The section shows the body of the tongue (asterisk) and the lingual-mandible sulcus (black arrows), with the mandibular bone (MB) observed laterally; (**b**) Fetus with microglossia and micrognathia: (b1) Frontal view of the head showing the triangular mouth smaller than the one from the control, due to a reduction of the mandible (M); (b2) The inferior view shows a smaller mandible (M), with a narrow arcade (dotted line); (b3) The section shows that the tongue is smaller, the muscles are disorganized (asterisk) and the lingual septum is absent; the lingual-mandible sulcus is enlarged (black arrows) and the mandibular bone (MB) is smaller; (**c**) Fetus with aglossia and agnathia: (c1) In a frontal view, the mouth is observed as an anterior to posterior cleft, due to the absence of the mandible (black arrow); (c2) The inferior view of the mouth is cleft-shaped (black arrow); (c3) In the section of the head, the tongue appears as a small bulge (asterisk), with absent or indistinguishable muscles (UM). If agnathia was concomitant, the mandible was also absent. Scale bar: 1 mm (a1, a2, b1, b2, c1 and c2); scale bar: 100 µm (a3, b3 and c3).

The main malformations observed affected the tongue as well as the mandible, as shown in (Figure 2). The tongue alterations were either microglossia (small tongue) or aglossia (absence of tongue). Likewise, the malformations observed in the mandible were micrognathia (small mandible) or agnathia (absence of mandible).

Aglossia was the most frequent malformation, affecting 16 fetuses (6.8% of the total; 69.6% of malformed fetuses), corresponding to six, eight, ten, 14 and 16 weeks of maternal FAD diet. Microglossia appeared in seven fetuses (3.0% of total, 30.4% of malformed fetuses), at eight, ten and 12 weeks of maternal FAD. The highest prevalence of malformations occurred in the offspring of females after 10 weeks of the FAD diet.

In the control fetuses, the mouth was visible as a small triangle in the anterior view and as a wide arcade in the inferior view (Figure 2(a1,2)). Sections showed the mandible present on both sides of the tongue, separated from it by a narrow lingual-mandible sulcus. The upper part of the tongue exceeded the body of the mandible and contacted the palate frequently. The intrinsic muscles of the tongue were clearly visible and the genioblossus and genihioid muscles formed a triangle partially divided by the lingual septum. These muscles were easily distinguishable at the base of the tongue, with the ducts of the salivary glands placed laterally (Figure 2(a3)).

In those animals suffering from microglossia, the mouth was also seen as a small triangle in the anterior view but the arcade was smaller and narrower in the inferior view. Sections revealed that the tongue was reduced in size, the muscles were present but disorganized, the lingual septum was total or partially disappeared, the lingual-mandibular sulcus was wider, and the mandibular bone was smaller. When a cleft palate was present, the tongue was always placed between the two halves of the palate. The mandible was reduced in size in all cases (micrognathia) (Figure 2(b1–3)).

Aglossia was also accompanied by micrognathia (four fetuses) or agnathia (12 fetuses). The extremely small or absent mandible caused the mouth to seem larger and cleft shaped in the anterior and inferior views. The sections showed that the tongue muscles were absent or indistinguishable, with no clear division between the right and left sides due to the lack of the lingual septum. In most of the cases, the tongue was represented by a small bulge, which did not extend beyond the dental arch (Figure 2(c1–3)).

When we studied the number of mothers who had fetuses with malformations of tongue gathering the weeks deficiency in FA, we observed that when mothers had more than two months on FAD diet the percentage of malformations in fetuses (56.5%) was significantly higher than the control mothers (0.0%) (*p* < 0.01, Fisher’s exact test).

### 3.2. Tongue and Head Measurements

**Figure 3:**
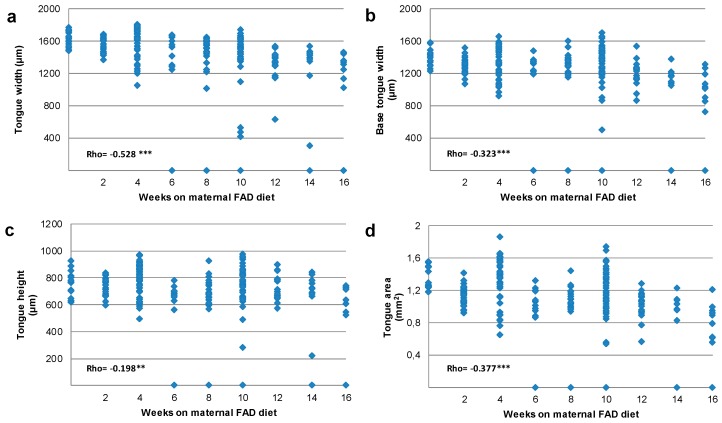
Correlation between tongue width (**a**); base of the tongue width (**b**); tongue height (**c**) and tongue area (**d**) with weeks on maternal FAD diet. ** *p* ≤ 0.01; *** *p* ≤ 0.001 Spearman’s Rho.

**Figure 4:**
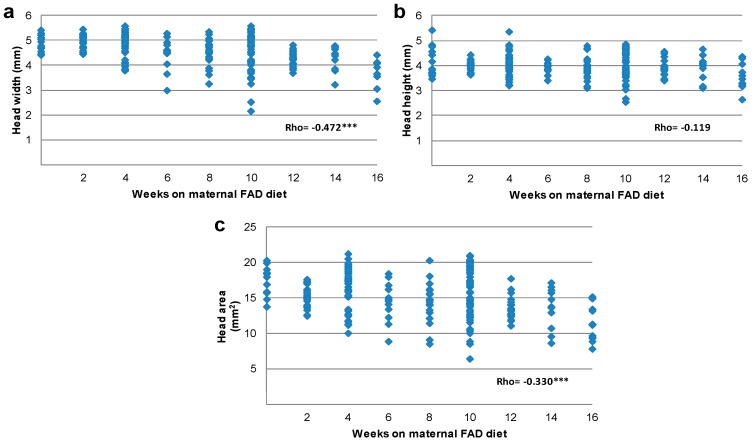
Correlation between head width (**a**); head height (**b**) and head area (**c**) with weeks on maternal FAD diet. *** *p* ≤ 0.001 Spearman’s Rho.

**Figure 5:**
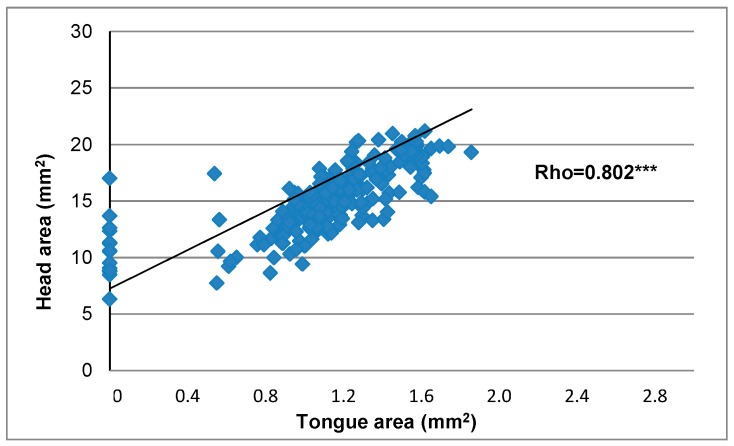
Correlation between head area and tongue area with weeks on maternal FAD diet. *** *p* ≤ 0.001 Spearman’s Rho.

A total of 225 fetal heads from control (*n* = 15) and experimental fetuses (*n* = 210) were analyzed to measure the tongue and head areas, (Table 3 and Table 4 and Figure 3, Figure 4 and Figure 5). The tongue width, base of the tongue body width and tongue area measurements were significantly reduced in individuals who had 16 maternal FAD diet weeks (Table 3) when compared to controls. The total reduction of the tongue measurement in maternal FAD diet fetuses was: 19.4% for tongue width; 25.8% for the base of the body of tongue width and 31.5% for the tongue area. Interestingly, tongue area was already reduced in the 6-week maternal FAD diet fetuses (Table 3).

Head standard measures started to decrease after 6 weeks of FAD diet with a significant reduction of the head area, which showed statistically significant lower growth after 10 weeks as compared to controls. This was the most affected measure, reaching a total reduction of 40% (Table 4). The least affected measure was the head height, which was not reduced significantly in FAD fetuses. The head width was significantly reduced from week 12 of FAD onwards and showed a total reduction of 26% (Table 4).

The correlation among head and tongue measures and weeks on maternal FAD diet (Figure 3 and Figure 4) showed an inverse significant association of the whole measures except for head height. The tongue and head width, tongue and head area, as well as the dorsum of tongue width were the most closely related to weeks on maternal FAD diet (*p* < 0.001 Spearman’s Rho). Finally, the correlation between tongue area and head area according to the number of weeks under maternal FAD diet presented a high statistical significance (Rho = 0.802), showing a harmonic and proportional reduction between the values of these structures (Figure 5).

In summary, the tongue and head measures were significantly reduced in the FAD fetuses, starting after six weeks of FAD diet. Likewise, the correlation between tongue and head measures related to weeks on maternal FAD diet showed a high statistical significance.

## 4. Discussion

The tongue is one of the major structures involved in human food intake and speech, although paradoxically it has been very scarcely studied. Tongue malformations such as aglossia, microglossia, and ankyloglossia are congenital birth defects greatly affecting individuals’ quality of life.

In this study, we show evidence, for the first time, that a maternal FA deficiency caused marked impairment of the tongue development, whereas an adequate maternal FA diet had powerful protective role in preventing tongue abnormalities.

The decline in maternal folate levels [21,25] was accompanied by tongue dysmorphologies starting at 6 weeks on maternal FAD diet, but when mothers had more than two months on FAD diet the percentage of malformations in fetuses (56.5%) was significantly higher than the control mothers (0.0%) (*p* < 0.01). In previous studies, we also observed a close temporal relationship between induced maternal folate deficiency and development of critical malformations in the eye and palate [21,25], the eyes being most affected with a 43.7% incidence [25].

The morphological and temporal differences between microglossia and aglossia we have found could indicate a dissimilar etiology of these malformations, as low levels of maternal FA have several possible modes of action on the development of tongue. 

A statistically significant linear association between the reduction of tongue and head areas and the number of maternal weeks on the FAD diet was observed, indicating that the reduction between these structures was harmonious and proportionate, which was consistent with the reduced embryonic growth that FA deficiency caused in the palate [21], tongue [27] or other organs [28].

Coordination and integration are key features during both early and late tongue and head development. The tongue develops from migrating cells from cranial NC which contribute to tongue connective tissue and vasculature, whereas most of the tongue muscles originate from myoblasts that have migrated from the occipital somites. The cranial NC cells populate the tongue primordium before the invasion of myogenic progenitors in mouse embryos, initiating and directing tongue development with reciprocal interactions between these two groups of cells [2,3,4,5,6]. 

It is critical that the embryo generates and maintains a sufficient pool of NC progenitors that survive, proliferate, migrate, and differentiate appropriately [29] as deficiencies in these processes underlie a number of congenital craniofacial malformation disorders as seen in a number of human syndromes with craniofacial malformations [30,31,32,33].

The alteration of cranial NC cells by FA has been previously established (see for review [34,35]). Folate is a nutrient which is known to impact DNA methylation due to its interaction with the one-carbon metabolism cycle. A decrease in the level of dietary folate has been found to decrease neonatal genomic DNA methylation levels in humans [30,32,34,35,36,37,38]. The molecular regulation of tongue myogenic progenitors involves different molecules for migration, proliferation, determination, differentiation and maturation with at least 10 genes working together in a time-bound collaboration cascade of the developing head. Among the top folate-associated genes is *STX11*, a gene critical for neural crest development. Reduced periconceptional folate intake was associated with increased methylation and, in turn, decreased gene expression at this loci [39].

The alteration of structures that derive from cranial NC cells, such as lingual vessels and the lingual septum observed in the aglossias and microglossias in this study suggests a defect of these cells. This is reinforced by the fact that these cells are the first to migrate to the future tongue, directing and coordinating the subsequent development of the myoblast population [2,4,6,9,21,28,29,40,41,42]. In consequence, we hypothesize that the lack of FA in the maternal diet alters the harmony of the genetic cascade required for proper formation of the tongue, with the NC cells mainly affected.

In humans, the development of the tongue begins when the human embryo is four weeks old. Malformations of the tongue are structural defects; the most common are aglossia, followed by microglossia, which is always combined with other defects forming different syndromes [43]. Agnathia-otocephaly is a rare, sporadic and lethal malformation characterized by microstomia (small mouth), aglossia, agnathia, and abnormally positioned ears. This complex disorder can be attributed to a failure of NC cells to migrate into the first and second pharyngeal arches which could cause dysplasia, hypoplasia, or even aplasia of the musculoskeletal derivatives from these arches, including the tongue [44]. For obvious reasons, an understanding of the etiology of these aberrations is fundamental for basic and translational science. 

Prevalence of marginal folate and vitamin B_12_ deficiency in western countries is increasing [45,46,47], but no parallel concern in society and public health policies has been observed. In Europe, it was shown that sub-clinical deficiency of folates and vitamin B6 could affect around 20% of European adolescents [48]. Likewise, the analysis of nutrient intake data from a review of a number of European countries showed a higher risk of inadequate folate intakes in adults and the elderly population/seniors when compared to the rest of the population [49]. Equally, the information of the European Nutrition and Health Report I provides an overview of folates inadequacy in European seniors [50]. Furthermore, Planells et al. [51] have given a precise estimate of the nutritional status for vitamins B6, B12 and folates in the adult population of southern Spain, and they have shown that factors such as age, place of residence, level of education and smoking habits can increase the risk of inadequate intake of these nutrients. Obviously, results from animal models to humans cannot be inferred, but this information may assist in suggesting future lines of research.

We believe that the present results strongly support the important role of adequate levels of folate during tongue development, and that its deficiency is relevant to the etiology of human congenital tongue abnormalities. The present morphological study of the structures affected by a maternal FAD diet during the complex formation of the tongue in mice is the starting point for future studies that provide insight into the altered mechanisms underlying this modification, with important potential consequences of functional and/or clinical relevance. 

## 5. Conclusions

This study demonstrates, for the first time, that an adequate folic acid/folate status plays a key role in the formation of the tongue and mandible, whereas a vitamin deficiency is negatively associated with normal tongue development. We therefore propose the tongue to be included in the list of FA-sensitive birth defect organs.

## Figures and Tables

**Table 1:** General outcome. Number of fetuses and type and incidence of malformation with respect to weeks on a maternal folic acid-deficient (FAD) diet.

Weeks on Maternal Fad Diet	Mothers (*n*)	Fetuses (*n*)	Tongue Malformation Fetuses *n* (% Total)	Microglossia Fetuses *n* (% Group; % Total)	Aglossia Fetuses *n* (% Group; % Total)
Control	5	25	0 (0)	0 (0.0; 0.0)	0 (0.0; 0.0)
2	4	29	0 (0)	0 (0.0; 0.0)	0 (0.0; 0.0)
4	7	46	0 (0)	0 (0.0; 0.0)	0 (0.0; 0.0)
6	4	13	2 (0.9)	0 (0.0; 0.0)	2 (100.0; 0.9)
8	5	25	4 (1.7)	1 (25.0; 0.4)	3 (75.0; 1.3)
10	9	54	12 (5.1)	4 (33.3; 1.7)	8 (66.7; 3.4)
12	5	21	2 (0.9)	2 (100.0; 0.9)	0 (0.0; 0.0)
14	2	11	2 (0.9)	0 (0.0; 0.0)	2 (100.0; 0.9)
16	2	11	1 (0.4)	0 (0.0; 0.0)	1 (100.0; 0.4)
Total	43	235	23 (9.8)	7 (30.4; 3.0)	16 (69.6; 6.8)

**Table 2:** General outcome. Number mothers with tongue malformation fetuses with respect to weeks on a maternal folic acid-deficient (FAD) diet.

		Weeks on Maternal FAD Diet			
		0	2–6	8–16	Total
**Mothers without tongue malformation fetuses**	***n***	5 ^a^	14 ^a^	10 ^b^	29
	**% group**	100.0	93.3	43.5	67.4
**Mothers with tongue malformation fetuses**	***n***	0 ^a^	1 ^a^	13 ^b^	14
	**% group**	0.0	6.7	56.5	32.6
**Total**	***n***	5	15	23	43
	**% group**	100.0	100.0	100.0	100.0

Each letter of the subscript denotes a subset of weeks categories whose column ratios do not differ significantly from each other at the 0.05.

**Table 3:** Effects of folate deficiency on tongue measurements.

Weeks on Maternal FAD Diet (n)	Tongue Width		Base of the Tongue Width		Tongue Height		Tongue Area	
	(μm)	VvC (%)	(μm)	VvC (%)	(μm)	VvC (%)	(mm^2^)	VvC (%)
Control	1629.7		1392.2		767.8		1.297	
(*n* = 15)	(1534.8–1717.6)		(1305.5–1453.4)		(702.0–816.0)		(1.243–1.500)	
2	1504.0	−7.7	1296.9 ***	−6.8	726.1	−5.4	1.042	−19.6
(*n* = 29)	(1602.3–1641.4)		(1296.9–1348.0)		(687.5–776.8)		(1.047–1.203)	
4	1688.0	3.6	1430.0	2.7	820.00	6.8	1.389	7.1
(*n* = 46)	(1505.6–1734.4)		(1225.0–1515.0)		(716.9–868.7)		(1.125–1.583)	
6	1412.9	−13.3	1239.0	−11.0	668.6	−12.9	1.013 **	−21.9
(*n* = 13)	(1266.2–1624.3)		(1195.2–1355.0)		(593.9–699.9)		(0.871–1.193)	
8	1504.9 *	−7.7	1321.3	−5.1	680.3	−11.4	1.051 **	−19.0
(*n* = 25)	(1287.0–1565.5)		(1203.0–1371.7)		(609.0–748.8)		(0.977–1.179)	
10	1499.5 *	−8.0	1274.9	−8.4	744.1	−3.1	1.147	−11.6
(*n* = 54)	(1393.5–1593.2)		(1174.2–1444.8)		(635.0–833.5)		(0.922–1.417)	
12	1386.0 *	−14.9	1252.5	−10.0	710.6	−7.5	1.029 **	−20.7
(*n* = 21)	(1298.2–1430.4)		(1127.3–1310.6)		(650.9–791.8)		(0.933–1.119)	
14	1392.9 **	−14.5	1166.6 *	−16.2	716.7	−6.7	0.969 **	−46.3
(*n* = 11)	(1171.0–1455.7)		(1077.8–1217.0)		(663.6–782.2)		(0.825–1.080)	
16	1314.3 ***	−19.4	1032.9 ***	−25.8	604.5	−21.3	0.888 ***	−31.5
(*n* = 11)	(1130.1–1360.1)		(856.9–1188.2)		(543.3–723.3)		(0.612–0.949)	

Values are median (interquartile range per group). * *p* < 0.05 vs. control group; ** *p* < 0.01 vs. control group, *** *p* < 0.001 vs. control group; (Kruskal–Wallis Test and the Dunn to adjust for multiple comparison and adjust the *p* value with Bonferroni correction). VvC: variation value relative to control.

**Table 4:** Effects of folate deficiency on head measurements.

Weeks of Maternal FAD Diet (*n*)	Head Width		Head Height		Head Area	
	(mm)	VvC (%)	(mm)	VvC (%)	(mm^2^)	VvC (%)
**Control**	5.0		3.9		17.9	
**(*n* = 15)**	(4.7–5.3)		(3.7–4.8)		(15.7–19.0)	
**2**	5.0	0.0	4.0	2.6	15.3	−14.5
**(*n* = 29)**	(4.7–5.2)		(3.7–4.1)		(14.2–16.1)	
**4**	5.0	0.0	4.1	5.1	17.2	−3.9
**(*n* = 46)**	(4.4–5.2)		(3.9–4.4)		(14.7–18.5)	
**6**	4.6	−8.0	4.0	2.6	14.7	−17.8
**(*n* = 13)**	(4.3–5.1)		(3.8–4.1)		(12.7–16.4)	
**8**	4.5	−10.0	3.8	−2.6	14.4	−19.5
**(*n* = 25)**	(4.3–4.8)		(3.7–4.1)		(13.6–15.8)	
**10**	4.7	−6.0	4.0	2.6	12.6 **	−29.6
**(*n* = 54)**	(3.8–5.1)		(3.7–4.5)		(15.3–18.9)	
**12**	4.3 **	−14.0	3.8	−2.6	13.3 **	−25.7
**(*n* = 21)**	(4.1–4.5)		(3.7–4.0)		(12.6–14.6)	
**14**	4.4	−12.0	4.0	2.6	13.7	−23.5
**(*n* = 11)**	(3.8–4.7)		(3.5–4.2)		(10.7–16.0)	
**16**	3.7 ***	−26.0	3.5	−10.2	11.1 ***	−40.0
**(*n* = 11)**	(3.6–4.1)		(3.2–4.1)		(9.2–13.4)	

Values are median (interquartile range per group). * *p* < 0.05 vs. control group; ** *p* < 0.01 vs. control group; *** *p* < 0.001 vs. control group; (Kruskal-Wallis Test and the Dunn to adjust for multiple comparison and adjust the *p* value with Bonferroni correction). VvC: variation value relative to control.

## Supplementary Materials

The following are available online at www.mdpi.com/2072-6643/10/1/26/s1.
