# Supplementary material for: Tongue Abnormalities Are Associated to a Maternal Folic Acid Deficient Diet in Mice
Source: Nutrients. 2017 Dec 28;10(1):26. doi: 10.3390/nu10010026 (PMC5793254; doi:10.3390/nu10010026)
Supplement: Supplementary file 1 [file nutrients-10-00026-s001.docx]

| Compplementary data | | |  |  |  |  |  |  |  |  |  |  |  |  |  |  |  |  |  |
| --- | --- | --- | --- | --- | --- | --- | --- | --- | --- | --- | --- | --- | --- | --- | --- | --- | --- | --- | --- |
| **Table 1. General outcome. Number and percentage of fetuses and type and incidence of malformations with respect to weeks on a maternal folic acid deficient (FAD) diet.  F: Fetuses. MF: Malformed fetuses.** | | | | | | | | | | | | | | | | | | | |
|  |  |  |  |  |  |  |  |  |  |  |  |  |  |  |  |  |  |  |  |
|  |  |  |  |  |  | MICROGLOSSIA | | | | | | | AGLOSSIA | | | | | | |
| Weeks on maternal FAD diet | Mothers (n) | F per group (n) | F per litter (n) | MF (n) | MF per group (n) | MF (n) | MF per litter (%) | MF per group (%) | Microglossias per group (%) | Total microglossias (n=7) (%) | Total MF (n=23) (%) | Total F (n=210) (%) | MF (n) | MF per litter (%) | MF per group (%) | Aglossias per group (%) | Total glossias (n=7) (%) | Total MF (n=23) (%) | Total F (n=210) (%) |
| Control | 5 | 25 | 5 | 0 | 0 | 0 | 0 | 0 | 0 | 0 | 0 | 0 | 0 | 0 | 0 | 0 | 0 | 0 | 0 |
|  |  |  |  |  |  |  |  |  |  |  |  |  |  |  |  |  |  |  |  |
|  |  |  |  |  |  |  |  |  |  |  |  |  |  |  |  |  |  |  |  |
| 2 | 4 | 29 | 7 | 0 | 0 | 0 | 0 | 0 | 0 | 0 | 0 | 0 | 0 | 0 | 0 | 0 | 0 | 0 | 0 |
|  |  |  | 8 | 0 |  | 0 | 0 | 0 | 0 | 0 | 0 | 0 | 0 | 0 | 0 | 0 | 0 | 0 | 0 |
|  |  |  | 7 | 0 |  | 0 | 0 | 0 | 0 | 0 | 0 | 0 | 0 | 0 | 0 | 0 | 0 | 0 | 0 |
|  |  |  | 7 | 0 |  | 0 | 0 | 0 | 0 | 0 | 0 | 0 | 0 | 0 | 0 | 0 | 0 | 0 | 0 |
|  |  |  |  |  |  |  |  |  |  |  |  |  |  |  |  |  |  |  |  |
| 4 | 7 | 46 | 2 | 0 | 0 | 0 | 0 | 0 | 0 | 0 | 0 | 0 | 0 | 0 | 0 | 0 | 0 | 0 | 0 |
|  |  |  | 8 | 0 |  | 0 | 0 | 0 | 0 | 0 | 0 | 0 | 0 | 0 | 0 | 0 | 0 | 0 | 0 |
|  |  |  | 3 | 0 |  | 0 | 0 | 0 | 0 | 0 | 0 | 0 | 0 | 0 | 0 | 0 | 0 | 0 | 0 |
|  |  |  | 7 | 0 |  | 0 | 0 | 0 | 0 | 0 | 0 | 0 | 0 | 0 | 0 | 0 | 0 | 0 | 0 |
|  |  |  | 9 | 0 |  | 0 | 0 | 0 | 0 | 0 | 0 | 0 | 0 | 0 | 0 | 0 | 0 | 0 | 0 |
|  |  |  | 9 | 0 |  | 0 | 0 | 0 | 0 | 0 | 0 | 0 | 0 | 0 | 0 | 0 | 0 | 0 | 0 |
|  |  |  | 8 | 0 |  | 0 | 0 | 0 | 0 | 0 | 0 | 0 | 0 | 0 | 0 | 0 | 0 | 0 | 0 |
|  |  |  |  |  |  |  |  |  |  |  |  |  |  |  |  |  |  |  |  |
| 6 | 4 | 13 | 4 | 0 | 2 | 0 | 0 | 0 | 0 | 0 | 0 | 0 | 0 | 0 | 0 | 0 | 0 | 0 | 0 |
|  |  |  | 1 | 0 |  | 0 | 0 | 0 | 0 | 0 | 0 | 0 | 0 | 0 | 0 | 0 | 0 | 0 | 0 |
|  |  |  | 3 | 0 |  | 0 | 0 | 0 | 0 | 0 | 0 | 0 | 0 | 0 | 0 | 0 | 0 | 0 | 0 |
|  |  |  | 5 | 2 |  | 0 | 0 | 0 | 0 | 0 | 0 | 0 | 2 | 40 | 15,4 | 100,0 | 12,5 | 8,7 | 1,0 |
|  |  |  |  |  |  |  |  |  |  |  |  |  |  |  |  |  |  |  |  |
| 8 | 5 | 25 | 5 | 2 | 4 | 1 | 20 | 4 | 25 | 14,3 | 4,3 | 0,5 | 1 | 20,0 | 4 | 25 | 6,25 | 4,3 | 0,5 |
|  |  |  | 4 | 0 |  | 0 | 0 | 0 | 0 | 0 | 0 | 0 | 0 | 0 | 0 | 0 | 0 | 0 | 0 |
|  |  |  | 7 | 1 |  | 0 | 0 | 0 | 0 | 0 | 0 | 0 | 1 | 14,3 | 4 | 25 | 6,25 | 4,3 | 0,5 |
|  |  |  | 4 | 1 |  | 0 | 0 | 0 | 0 | 0 | 0 | 0 | 1 | 25,0 | 4 | 25 | 6,25 | 4,3 | 0,5 |
|  |  |  | 5 | 0 |  | 0 | 0 | 0 | 0 | 0 | 0 | 0 | 0 | 0 | 0 | 0 | 0 | 0 | 0 |
|  |  |  |  |  |  |  |  |  |  |  |  |  |  |  |  |  |  |  |  |
| 10 | 9 | 54 | 7 | 1 | 12 | 0 | 0 | 0 | 0 | 0 | 0 | 0 | 1 | 14,3 | 1,9 | 8,3 | 6,3 | 4,3 | 0,5 |
|  |  |  | 10 | 0 |  | 0 | 0 | 0 | 0 | 0 | 0 | 0 | 0 | 0 | 0 | 0 | 0 | 0 | 0 |
|  |  |  | 6 | 1 |  | 0 | 0 | 0 | 0 | 0 | 0 | 0 | 1 | 16,7 | 1,9 | 8,3 | 6,3 | 4,3 | 0,5 |
|  |  |  | 9 | 4 |  | 2 | 22,2 | 3,7 | 16,7 | 28,6 | 8,7 | 1,0 | 2 | 22,2 | 3,7 | 16,7 | 12,5 | 8,7 | 1,0 |
|  |  |  | 4 | 0 |  | 0 | 0 | 0 | 0 | 0 | 0 | 0 | 0 | 0 | 0 | 0 | 0 | 0 | 0 |
|  |  |  | 6 | 0 |  | 0 | 0 | 0 | 0 | 0 | 0 | 0 | 0 | 0 | 0 | 0 | 0 | 0 | 0 |
|  |  |  | 2 | 2 |  | 0 | 0 | 0 | 0 | 0 | 0 | 0 | 2 | 100,0 | 3,7 | 16,7 | 12,5 | 8,7 | 1,0 |
|  |  |  | 6 | 3 |  | 2 | 33,3 | 3,7 | 16,7 | 28,6 | 8,7 | 1,0 | 1 | 16,7 | 1,9 | 8,3 | 6,3 | 4,3 | 0,5 |
|  |  |  | 4 | 1 |  | 0 | 0 | 0 | 0 | 0 | 0 | 0 | 1 | 25,0 | 1,9 | 8,3 | 6,3 | 4,3 | 0,5 |
|  |  |  |  |  |  |  |  |  |  |  |  |  |  |  |  |  |  |  |  |
| 12 | 5 | 21 | 3 | 0 | 2 | 0 | 0 | 0 | 0 | 0 | 0 | 0 | 0 | 0 | 0 | 0 | 0 | 0 | 0 |
|  |  |  | 7 | 1 |  | 1 | 14,3 | 4,8 | 50,0 | 14,3 | 4,3 | 0,5 | 0 | 0 | 0 | 0 | 0 | 0 | 0 |
|  |  |  | 5 | 0 |  | 0 | 0 | 0 | 0 | 0 | 0 | 0 | 0 | 0 | 0 | 0 | 0 | 0 | 0 |
|  |  |  | 4 | 1 |  | 1 | 25,0 | 4,8 | 50,0 | 14,3 | 4,3 | 0,5 | 0 | 0 | 0 | 0 | 0 | 0 | 0 |
|  |  |  | 2 | 0 |  | 0 | 0 | 0 | 0 | 0 | 0 | 0 | 0 | 0 | 0 | 0 | 0 | 0 | 0 |
|  |  |  |  |  |  |  |  |  |  |  |  |  |  |  |  |  |  |  |  |
| 14 | 2 | 11 | 7 | 2 | 2 | 0 | 0 | 0 | 0 | 0 | 0 | 0 | 2 | 28,6 | 18,2 | 100,0 | 12,5 | 8,7 | 1,0 |
|  |  |  | 4 | 0 |  | 0 | 0 | 0 | 0 | 0 | 0 | 0 | 0 | 0 | 0 | 0 | 0 | 0 | 0 |
|  |  |  |  |  |  |  |  |  |  |  |  |  |  |  |  |  |  |  |  |
| 16 | 2 | 11 | 8 | 0 | 1 | 0 | 0 | 0 | 0 | 0 | 0 | 0 | 0 | 0 | 0 | 0 | 0 | 0 | 0 |
|  |  |  | 3 | 1 |  | 0 | 0 | 0 | 0 | 0 | 0 | 0 | 1 | 33,3 | 9,1 | 100,0 | 6,3 | 4,3 | 0,5 |
| Total | 41 |  | 225 | 23 |  | 7 |  |  |  |  | 30,4 | 3,3 | 16 |  |  |  |  | 69,6 | 7,6 |
| FAD | 38 |  | 210 | 23 |  | 7 |  |  |  |  | 30,4 | 3,3 | 16 |  |  |  |  | 69,6 | 7,6 |
